# Characterization of an inducible promoter in different DNA copy number conditions

**DOI:** 10.1186/1471-2105-13-S4-S11

**Published:** 2012-03-28

**Authors:** Susanna Zucca, Lorenzo Pasotti, Giuliano Mazzini, Maria Gabriella Cusella De Angelis, Paolo Magni

**Affiliations:** 1 Dipartimento di Informatica e Sistemistica, Università degli Studi di Pavia, Via Ferrata 1, I-27100 Pavia, Italy; 2 Centro di Ingegneria Tissutale, Università degli Studi di Pavia, Via Ferrata 1, I-27100 Pavia, Italy; 3 Istituto di Genetica Molecolare - Consiglio Nazionale delle Ricerche, Via Ferrata 9, I-27100 Pavia, Italy

## Abstract

**Background:**

The bottom-up programming of living organisms to implement novel user-defined biological capabilities is one of the main goals of synthetic biology. Currently, a predominant problem connected with the construction of even simple synthetic biological systems is the unpredictability of the genetic circuitry when assembled and incorporated in living cells. Copy number, transcriptional/translational demand and toxicity of the DNA-encoded functions are some of the major factors which may lead to cell overburdening and thus to nonlinear effects on system output. It is important to disclose the linearity working boundaries of engineered biological systems when dealing with such phenomena.

**Results:**

The output of an N-3-oxohexanoyl-L-homoserine lactone (HSL)-inducible RFP-expressing device was studied in *Escherichia coli *in different copy number contexts, ranging from 1 copy per cell (integrated in the genome) to hundreds (via multicopy plasmids). The system is composed by a luxR constitutive expression cassette and a RFP gene regulated by the luxI promoter, which is activated by the HSL-LuxR complex. System output, in terms of promoter activity as a function of HSL concentration, was assessed relative to the one of a reference promoter in identical conditions by using the Relative Promoter Units (RPU) approach. Nonlinear effects were observed in the maximum activity, which is identical in single and low copy conditions, while it decreases for higher copy number conditions. In order to properly compare the luxI promoter strength among all the conditions, a mathematical modeling approach was used to relate the promoter activity to the estimated HSL-LuxR complex concentration, which is the actual activator of transcription. During model fitting, a correlation between the copy number and the dissociation constant of HSL-LuxR complex and luxI promoter was observed.

**Conclusions:**

Even in a simple inducible system, nonlinear effects are observed and non-trivial data processing is necessary to fully characterize its operation. The in-depth analysis of model systems like this can contribute to the advances in the synthetic biology field, since increasing the knowledge about linearity and working boundaries of biological phenomena could lead to a more rational design of artificial systems, also through mathematical models, which, for example, have been used here to study hard-to-predict interactions.

## Background

Synthetic biology aims at implementing novel and user-defined capabilities in living systems that could yield applications of remarkable importance, like bioremediation or production of renewable fuels, new biomaterials and therapeutic molecules [1-3]. Engineering plays a crucial role in the rational design and construction of such systems, as principles like standardization, modularity and predictability of biological parts are considered key aspects [4]. The synthetic biology paradigm can be summarized as follows: i) choose biological parts from a library of well-characterized standard DNA components; ii) assemble them together to obtain a genetic program that encodes the desired function; iii) incorporate it in a living organism to ultimate the job, as it is carried out in many fields of engineering. Although the development of DNA standards (such as the BioBrick™in the Registry of Standard Biological Parts) [5,6] and automated DNA assembly platforms [7,8] have significantly reduced the complexity of the genetic program composition process, each of the mentioned steps hides noteworthy difficulties [9,10]: inability to provide reproducible quantitative characterization of parts [11], crosstalk or incompatibility among components [1], time-consuming debugging of systems [12], variability connected with biological processes [13] and, finally, a high failure rate of the engineered organism due to DNA mutation [14]. Taken together, these points contribute to the inability to predict the behaviour of even simple synthetic biological systems. In fact, although relatively complex systems such as bistable switches [15], oscillators [16,17] logic functions [18-22], amplifiers [23] have been successfully built up in bacteria, yeasts or mammalian cells and a variety of computational tools have been developed to aid their design [24-26], the construction of predictable biological systems from the bottom-up is currently a main challenge [9,27]. Trial-and-error or directed evolution screenings are still commonly used to optimize and even repair suboptimal genetic circuits [18,28-30]. Incorporation of genetic programs in a host, such as a bacterial cell, can be performed either through plasmid vectors, which are able to autonomously replicate in the organism and propagate the DNA-encoded synthetic functions to the progeny [31,32], or through genomic integration in which the program is stably kept in the cell in single copy [33]. Plasmids are maintained in cells at a copy number ranging from 1 or 2 to hundreds of copies, depending on their replication origin [34].

**Figure 1:**
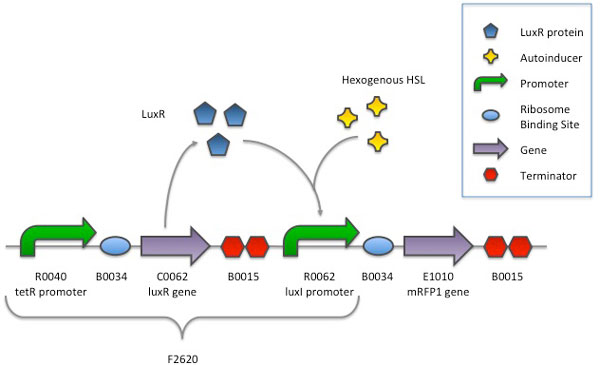
**Working diagram of the HSL-inducible system**. The luxR gene is constitutively produced by the tetR promoter and the strong ribosome binding site B0034. The gene encoding for mRFP1 is placed downstream of the luxI promoter. LuxR protein is normally unactive, but when HSL is present in the culture it binds LuxR and a complex is formed by two molecules of LuxR and two of HSL. This complex triggers the transcription of the luxI promoter in a concentration-dependent fashion.

In the literature, several mathematical models based on the differential equations have been proposed to describe the output of a synthetic circuit, often in terms of a synthesized mRNA or protein amount, as a function of its DNA copy number [35-37]. Considering the simplest system composed by one promoter and one gene of interest downstream, its dynamic behaviour is governed by the following equations which include transcription and translation processes [38]:

(1) $$\frac{d\left[M\left(t\right)\right]}{dt}=n\cdot r\left(t\right)-d\cdot \left[M\left(t\right)\right]$$

(2) $$\frac{d\left[P\left(t\right)\right]}{dt}=\rho \cdot \left[M\left(t\right)\right]-\gamma \cdot \left[P\left(t\right)\right]$$

where squared brackets indicate a per-cell concentration, *n *is the copy number of the DNA, *M *is the mRNA, *P *is the protein, *r *is the mRNA synthesis rate per DNA copy, *ρ *is the protein synthesis rate per mRNA and *d *and *γ *are the degradation rates of *M *and *P*, respectively. Assuming the steady state, it results that $\left[\bar{M}\right]=\frac{n\bar{r}}{d}$ and $\left[\bar{P}\right]=\frac{n\rho \bar{r}}{d\gamma }$, where bar indicates that the species is at the steady state (constant value). Even if these expressions show that both mRNA and protein concentrations are theoretically linear functions of n, a number of works report that both transcription and translation output may not change linearly with the DNA copy number and yield hard-to-predict system outputs [28,39-41]. In particular, Hajimorad et al. [39] showed that the mRNA level of one or more gene expression devices changes linearly with the device copy number, but only in specific conditions, i.e. limited copy number and number of different devices in the same cell. In general, all the genetic manipulations that cause host overburdening may contribute to nonlinear effects on biological systems. It is also known that highly expressed recombinant genes can lead to saturation effects, caused by the overloading of endogenous transcriptional and translational machinery, partly for the limited availability of RNA polymerases and ribosomes [28,42]. In this work, an inducible promoter, which can be regulated over a wide range of transcriptional activities, was studied in *Escherichia coli *in single- and multi-copy contexts. The system is based on the widely studied luxR/luxI promoter system [37,43] and it is able to produce a red fluorescent protein (RFP) upon N-3-oxohexanoyl-L-homoserine lactone (HSL) molecule addition to the bacterial culture in a concentration-dependent fashion (see Figure 1 for the inducible system description). The DNA encoding the inducible system was placed in the bacterial genome or in plasmids with the pSC101, p15A or the mutated pMB1 replication origins, which yield low, medium or high copy numbers, respectively. The number of DNA copies per cell has been previously reported to be ~5, 20-30 and >100 for these three origins [34,44]. The relative promoter unit (RPU) approach [36] was used to indirectly measure the activity of the luxI promoter from RFP fluorescence data for each investigated induction and device copy number context. RPU is a method in which the strength of a promoter is measured relative to the activity of BBa_J23101 constitutive promoter, chosen in the literature as a standard reference, with identical reporter gene, ribosome binding site (RBS) and plasmid [36]. In such framework, it is possible to investigate if the ratio between the outputs of synthetic devices, in terms of synthesized RFP, is maintained as a function of induced promoter strength and in different copy-number contexts. The results produced in this work can contribute to improve the characterization of a simple but widely used genetic device and such findings can represent a step towards the study of linearity boundaries of synthetic biology components when incorporated in living cells, thus enabling a more rational design of biological systems. An in-depth experimental analysis of such nonlinearities may also contribute to the advances in the field of systems biology, as increasing the knowledge about the working conditions of biological systems could lead to the creation of new mathematical models of higher accuracy, useful to computationally study or predict complex biological phenomena through simulations.

## Methods

### Strains and plasmids

The description of all the *Escherichia coli *strains, vector backbones and genetic devices used in this work is shown in Table 1. The description of plasmids assembly steps is reported in the Additional file 1 (Plasmid construction). All the plasmids containing ccdB toxin were cloned in the ccdB-tolerant strain DB3.1. All the conditional-replication plasmids (p*ϕ*80 series) were cloned in the pir-116 strain BW23474. TOP10 strain was used to clone all the other plasmids. MG1655 strain was used as a host for all the quantitative experiments of this study.

**Table 1:** Strains, plasmids and biological devices used in this work.

Strains			
**Name**	**Genotype**		**Source**

DB3.1	F- gyrA462 endA1 glnV44 Δ(sr1-recA) mcrB mrr hsdS20 $\left({\text{r}}_{B}^{-},{\text{m}}_{B}^{-}\right)$ ara14 galK2 lacY1 proA2 rpsL20(Sm*^r^*) xyl5 Δleu mtl1		Invitrogen
BW23474	F-, Δ(argF-lac)169, ΔuidA4::pir-116, recA1, rpoS396(Am), endA9(del-ins)::FRT, rph-1, hsdR514, rob-1, creC510		CGSC, Yale University, USA
TOP10	F- mcrA Δ(mrr-hsdRMS-mcrBC) *ϕ*80lacZΔM15 ΔlacX74 nupG recA1 araD139 Δ(ara-leu)7697 galE15 galK16 rpsL(Str*^R^*) endA1 *λ*-		Invitrogen
MG1655	F- *λ*-ilvG- rfb-50 rph-1		CGSC Yale University, USA
MG-HSL*_RFP _*	MG1655, Φ80(HSL*_RFP _*)		This study
MG-101*_RFP _*	MG1655, Φ80(101*_RFP_*)		This study
MG-IQ*_RFP _*	MG1655, Φ80(IQ*_RFP_*)		This study

**Vector backbones used for RFP expression or genomic integration of the devices**			

Name	Replication origin	BioBrick™vector code	Antibiotic resistance

pHC	pUC19-derived pMB1 origin (high copy)	pSB1A2	Ampicillin
pMC	pMR101-derived p15A origin (medium copy)	pSB3K3	Kanamycin
pLC	pSC101 origin (low copy)	pSB4C5	Chloramphenicol
pΦ80	R6K conditional origin, used for integration	BBa_K300000	Chloramphenicol

**Genetic devices**			

Name	Description		

HSL*_RFP _*	HSL-inducible mRFP1 expression system		
HSL*_GFP _*	HSL-inducible GFPmut3b expression system		
101*_RFP _*	Standard reference constitutive promoter with mRFP1 expression device downstream		
101*_GFP _*	Standard reference constitutive promoter with GFPmut3b expression device downstream		
IQ*_RFP _*	lacIQ constitutive promoter with mRFP1 expression device downstream		

### Cloning methods

Chemically competent TOP10 and DB3.1 were used according to manufacturer's instructions. Chemically competent MG1655 and BW23474 were prepared according to [45] and were transformed by heat shock at 42°C. All the strains were routinely grown in a 5-ml volume of LB medium [45] at 37°C and 220 rpm. When required, Ampicillin (100 mg/L), Kanamycin (20 mg/L) or Chloramphenicol (12.5 mg/L) were added to cultures to maintain plasmids. Long-term glycerol stocks, routinely stored at -80°C, were prepared for all the recombinant strains by mixing 750 *μ*l of bacterial culture and 250 *μ*l of sterile 80% glycerol. Plasmids were extracted from overnight cultures through NucleoSpin Plasmid kit (Macherey-Nagel). DNA was digested as appropriate and the fragments of interest were extracted from 1% agarose gel by NucleoSpin Extract II kit (Macherey-Nagel) before proceeding with ligation to ultimate the assembly. DNA-modifying enzymes were purchased from Roche Diagnostics and used according to manufacturer's instructions. DNA sequencing of plasmids and PCR products was performed through BMR Genomics (Padova, Italy) DNA analysis service.

### Integrant strains

MG-HSL*_RFP_*, MG-101*_RFP _*and MG-IQ*_RFP _*were obtained by integrating HSL*_RFP_*, 101*_RFP _*and IQ*_RFP _*into the MG1655 host (through p*ϕ*80-HSL*_RFP_*, p*ϕ*80-101*_RFP _*and p*ϕ*80-IQ*_RFP _*integrative plasmids, respectively), as described in the BBa_K300000 integrative vector page in the Registry of Standard Biological Parts [46]. After genomic integration (mediated by the pInt80-649 helper plasmid [2]) and marker excision (mediated by the pCP20 helper plasmid [47]), only one copy of the expression device of interest remains in the *E. coli *chromosome and it is flanked by four transcriptional terminators to achieve isolation from genomic context. No antibiotic resistance is present at the end of the procedure, so the resulting recombinant strains are always grown in nonselective medium. Integrants were PCR-verified with primers P1 (5'-CTGCTTGTGGTGGTGAAT-3') and P4 (5'-TAAGGCAAGACGATCAGG-3'), annealing in the genome [33]. Platinum Taq DNA polymerase (Invitrogen) was used for PCR. When required, amplified fragments were gel-extracted as described above and sequenced.

### Population-based fluorescence assays

Recombinant strains were grown at 37°C, 220 rpm for about 16 hours in selective M9 supplemented medium (11.28 g/L M9 salts, 1 mM thiamine hydrochloride, 2 mM MgSO4, 0.1 mM CaCl2, 0.2% casamino acids and 0.4% glycerol as carbon source) [45], inoculated with a single colony from a streaked selective LB-agar plate. The cultures were 100-fold diluted in fresh selective medium and grown under the same conditions for 6 hours, then they were diluted at the optical density at 600 nm (OD_600_) of 0.03 and grown for 45 minutes. For the cultures that did not need to be induced, 200 *μ*l were transferred into a flat-bottomed 96-well microplate (Greiner) and incubated in the Infinite F200 (Tecan) reader. For the cultures bearing the HSL-inducible system, 200 *μ*l for each investigated HSL concentration were transferred and 2 *μ*l of properly diluted HSL (Sigma Aldrich) were added to the culture before incubation to yield the desired concentration. A kinetic cycle, programmed with the i-control™software (Tecan), was used to assay the cultures: every 5 min, 15 s linear shaking (3 mm amplitude), 5 s wait, absorbance measurement, fluorescence measurement were performed. RFP detection was carried out with 535/620 nm filters for excitation/emission and acquisition gain as appropriate, ranging from 50 to 100. GFP-expressing cells were assayed through the same procedure, but using the 485/540 nm filters for fluorescence acquisition. Together with the cultures of interest, 200 *μ*l of M9 supplemented medium and a non-fluorescent MG1655 culture were also included in each experiment to measure the background of absorbance and fluorescence respectively. A preliminary evaluation of the measurement system was carried out and results are reported in the Additional file 1 (Validation of the measurement system).

### Single-cell experiments

Recombinant strains expressing RFP or GFP were grown as described above and induced with HSL when required. After 2/3 hours from the induction, cells were either spread on a glass slide (for microscopy analysis) or properly diluted in sterile PBS (for flow cytometric analysis).

RFP-expressing cells were analyzed with an Olympus BX51 microscope with standard fluorescence equipment (HBO100W/2 lamp). Microphotographs were taken using an Olympus Camedia C-4040 digital camera. Fluorescence was detected through the green excitation performed with a 530-560 nm band pass excitation filter and a 590 nm dichroic mirror combined with a long pass barrier filter at 620 nm. UPlanFl 40× objective was used for all the acquisitions.

GFP-expressing cells were analyzed through a Partec PAS II flow cytometer equipped with an argon ion laser using the 488 nm blue line for excitation. Fluorescence emission was collected in FL1 by means of a 515-545 nm band pass filter. 100,000 events were collected and stored for each sample.

### Data analysis

Raw absorbance and fluorescence time series were processed with the MATLAB 2007b suite (MathWorks, Natick, MA) to obtain doubling time, average RFP synthesis rate per cell (*S_cell_*) and RPU during the exponential growth phase for each culture. The background absorbance time series of M9 supplemented medium was subtracted from each culture of interest to obtain the actual OD_600 _of bacterial cells.

Similarly, the fluorescence background time series was subtracted from the raw fluorescence of each culture to yield the actual RFP fluorescence of the bacterial population in the microplate well. Exponential growth phase was identified by visual inspection as the linear region of the *ln*(*OD*_600_) time series and the slope *m *of this line was computed with linear regression to yield the cell growth rate. Doubling time was computed as *ln*(2)/*m*. A signal proportional to the RFP synthesis rate per cell was computed as the numeric time derivative of RFP time series, divided by OD_600 _over time. This signal was averaged over the exponential growth phase, starting after 50 min from the induction time, to obtain the *S_cell _*of a culture [37,48]. *S_cell _*value is expressed in Arbitrary Units of RFP per minute per cell (AU min^-1 ^cell^-1^) at a specific gain, which represent an absolute unit [36]. Relative units were computed as *RPU*_*x *_= *S*_*cell*,*x*_/*S*_*cell, ref*_, where *x *is the culture of interest and *ref *is the culture bearing the standard reference promoter with RFP (101*_RFP _*), in the same copy number conditions as the culture of interest and acquired with the same gain factor [36]. Data analysis for GFP-expressing cells was conducted with the same procedure. Induction curves were fitted with the Hill equation $Y=V_{max}*\frac{I^{q}}{{K_{m}}^{q}+I^{q}}$, where *V_max _*is the maximum activity, *Y *can be either the *S_cell _*or the RPU of a system and *I *can be either the HSL concentration or the intracellular level of the HSL-LuxR activator complex, depending on the specific application. Basal activity of the luxI promoter was omitted from the Hill equation because its entity was much lower than *V_max _*in all the curves. The least squares fitting was performed through the MATLAB *lsqnonlin *routine. Among the presented results, *S_cell _*were shown for a set of cultures expressing RFP. Because a wide range of fluorescence intensities is produced by the studied cultures and then to have a good sensitivity in the acquisition process the fluorescence acquisition gain was tuned accordingly, *S_cell _*results had to be reported to the same gain (in this work a gain = 50 was chosen) to enable comparisons among values. To this aim, the fluorescence of a MG1655 culture bearing pHC-HSL*_RFP_*, grown for 6 hours in presence of 100 nM of HSL and diluted to OD_600 _= 0.01, was measured with different gain factors (50, 60, 70, 80, 90 and 100), set via i-control™software. All the collected measurements were divided by the RFP raw value at gain = 50, to compute RFP_*norm*,50 _and a calibration curve of gain vs RFP_*norm*,50 _was obtained (data not shown). Thus, *S_cell _*values computed from data acquired at gain = *β *were reported to gain = 50 by dividing them by the right conversion factor between *β *and 50, i.e. the RFP_*norm*,50 _value of the calibration curve corresponding to gain *β*.

Bright-field pictures acquired from the microscope were processed through the ImageJ software (Wayne Rasband, NIH) to enhance the contrast of photographed cells when required. Fluorescence images were not processed in any way.

Flow cytometric data analysis was carried out with the FloMax (Partec, Munster, Germany) software.

### Mathematical modeling

The following set of differential equations was used to describe the dynamics of LuxR (*X*), HSL-LuxR activated complex (*A*) and RFP (*R*) as a function of HSL (*H*) in the HSL-inducible system:

(3) $$\frac{dX}{dt}=n\cdot \alpha_{tet}-\gamma_{X}\cdot X$$

(4) $$A=\frac{X}{1+{\left(\frac{K_{H}}{H}\right)}^{n_{H}}}$$

(5) $$\frac{dR}{dt}=\frac{n\cdot \alpha_{lux}}{1+{\left(\frac{K_{A}}{A}\right)}^{n_{A}}}-\mu \cdot R$$

The description of the species and parameters is reported in Table 2. Parameter values that have not been estimated in this study are described in [48]. The degradation rate of RFP is much lower than the cell growth rate, so only the dilution caused by cell division contributes to the intracellular extinction of RFP (results not shown). On the other hand, dilution rate was neglected for LuxR when compared to the protein degradation rate [48]. The observable (measured) variable in each experiment is the RFP synthesis rate per cell, $S_{cell}=\frac{n\alpha_{lux}}{1+{\left(\frac{K_{A}}{A}\right)}^{n_{A}}}$. This is measured at the steady state and the maturation dynamics of the fluorescent protein [38] was not taken into account. Considering the steady state, $\bar{X}=\frac{n\alpha_{tet}}{\gamma_{X}}$, $Ā=\frac{\bar{X}}{1+{\left(\frac{K_{H}}{H}\right)}^{n_{H}}}$ and ${\bar{S}}_{{}_{cell}}=\frac{n\alpha_{lux}}{1+{\left(\frac{K_{A}}{A}\right)}^{n_{A}}}$, where the bar indicates that the species is at the steady state. Model fitting was performed from RPU data points instead of *S_cell _*to improve the reliability of the measured data. In particular, the *α_lux _*parameter was computed by multiplying the RPUs at full induction (indicated with *V_max_*) by the $S_{cell,101_{RFP}}/n$ term, where $S_{cell,101_{RFP}}$ is referred to the 101*_RFP _*device at the copy number *n*.

**Table 2:** Species and parameters included in the mathematical model.

Parameter or species	Description	Value	Units
*X*	LuxR protein concentration per cell	Variable	AU cell^-1^
*A*	LuxR-HSL activated complex concentration per cell	Variable	AU cell^-1^
*R*	RFP per cell	Variable	AU cell^-1^
*H*	Inducer concentration	Known input	nM
*n*	DNA copy number	Estimated	-
*α_tet_*	LuxR synthesis rate per cell per DNA copy	2.3^(*a*)^	AU min^-1 ^cell^-1^
*α_lux_*	Maximum RFP synthesis rate per cell per DNA copy	Estimated	AU min^-1 ^cell^-1^
*γ_X_*	LuxR protein degradation rate	6*10^-2^	min^-1^
*μ*	Cell growth rate	Estimated	min^-1^
*K_H_*	Dissociation constant of HSL-LuxR	553	nM
*K_A_*	Dissociation constant of HSL-LuxR complex and luxI promoter	Estimated	AU cell^-1^
*n_H_*	Hill cooperativity constant of HSL-LuxR	2	-
*n_A_*	Hill cooperativity constant of HSL-LuxR complex and luxI promoter	Estimated	-

^(*a*) ^From unpublished data of our lab.

Parameter values that have not been estimated in this study are described in [48]. AU indicates arbitrary units of RFP.

## Results

### Per-cell fluorescence of constitutive RFP-producing systems as a function of copy number

In order to show that variations in the copy number in an RFP-producing system cause different fluorescence levels, *S_cell _*was measured in the 101*_RFP _*and IQ*_RFP _*devices. Table 3 reports the resulting average values, which demonstrate that a wide range of synthesis rate can be achieved in the different conditions. *S_cell _*varies more than 100-fold between the two extreme conditions and increases with the DNA copy number as expected. All the *S_cell _*values were divided by the corresponding value measured in single copy, thus obtaining a rough and indirect estimation of the DNA copy number per cell [44]. The computed values are reported in Table 3. They are consistent with previously published copy number results measured in *E. coli *for the same replication origins, except for the medium copy which is about 2-fold higher than expected for a p15A replication origin.

**Table 3:** Characterization of J23101 and lacIQ promoters in absolute units and indirect copy number estimation

*S_cell_*[*AUcell*^-1^*min*^-1^]				
	HC	MC	LC	SC

101*_RFP_*	88.36	34.2	2.48	0.69
*IQ_RFP_*	59.1	22.7	2.14	0.54

**Estimated copy number**				

101*_RFP_*	128	49	4	1
*IQ_RFP_*	110	42	4	1

Measurement of the absolute activity of J23101 and lacIQ constitutive promoters via the 101*_RFP _*and *IQ_RFP _*devices and indirect copy number estimation from average *S_cell _*values. AU indicates arbitrary units of RFP.

### Induction curves of the HSL-inducible system in different copy number conditions as a function of HSL

**Figure 2:**
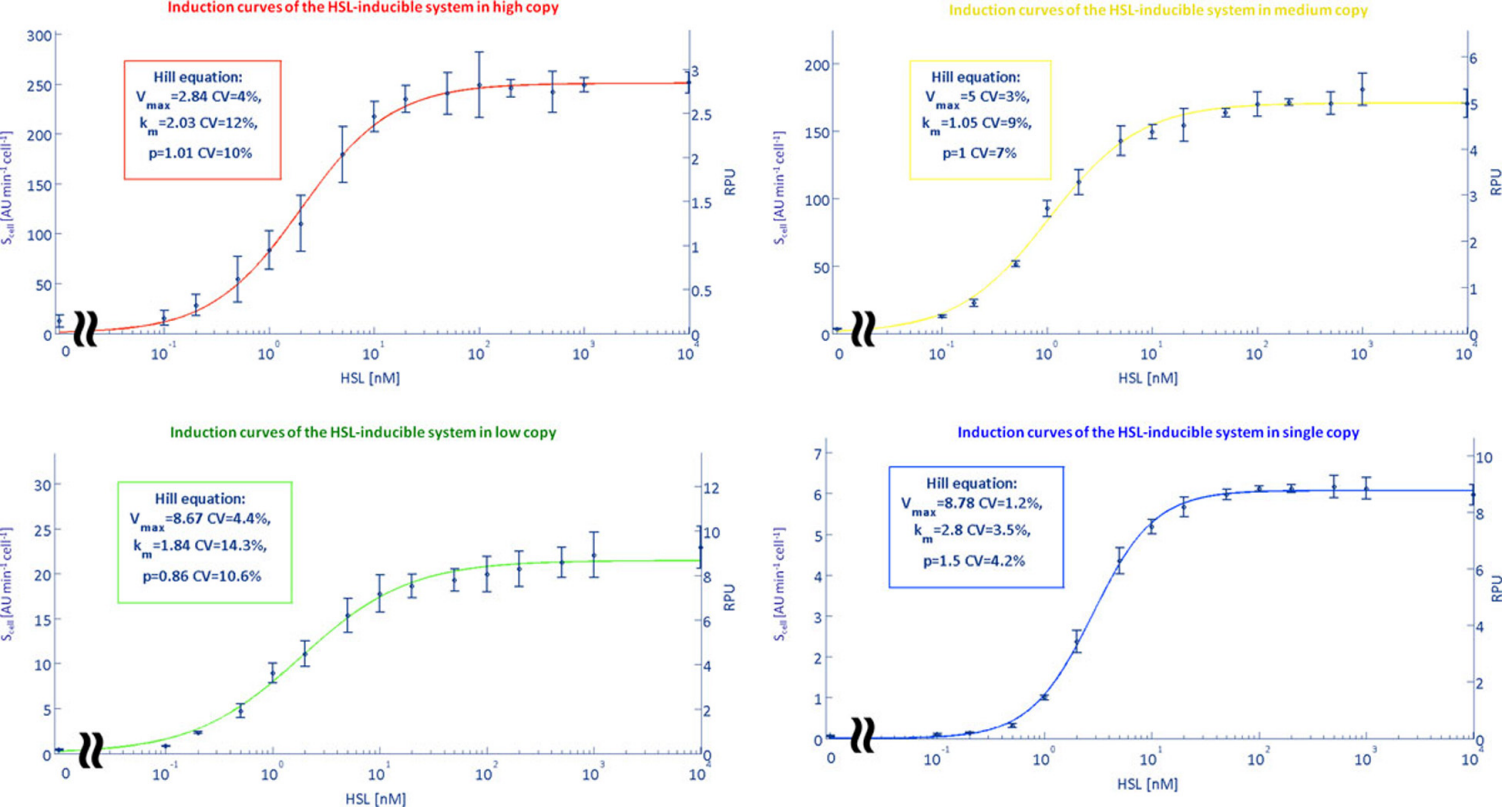
**Induction curves for the HSL-inducible system in different copy number contexts**. The HSL-inducible device (*HSL_RFP _*) was characterized in the exponential phase after at least 50 minutes from the induction in a microplate reader. For each copy number condition, the curve is expressed in absolute arbitrary units of *S_cell _*(left vertical axis) and in RPUs (right vertical axis), computed as the ratio between the *S_cell _*of the inducible device and the *S_cell _*of the J23101 promoter (via the 101*_RFP _*measurement device) in the same growth and copy number condition, considering the same reporter gene (RFP). Experimental data were fitted with a Hill function (continuous line) and the estimated parameters are reported in the boxes with their coefficients of variation. Error bars represent the 95% confidence intervals of the mean value (circles) computed on 3 clones.

**Figure 3:**
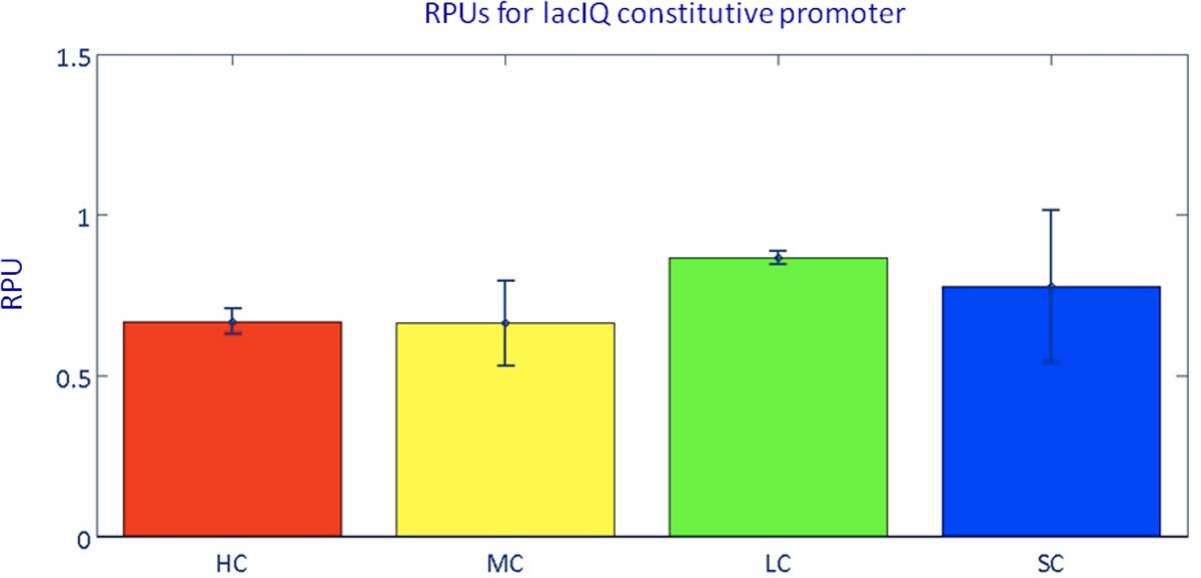
**Characterization of the lacIQ constitutive promoter in RPUs**. The ratio between the activities (*S_cell_*) of lacIQ and J23101 promoters, measured via *IQ_RFP _*and 101*_RFP _*devices respectively, was computed to obtain the lacIQ RPUs in the four copy number conditions investigated in this work. Consider that *IQ_RFP _*and 101*_RFP _*have a different DNA scar between promoter and RBS. Unpublished data from our lab showed that the scar present in *IQ_RFP _*systematically overestimates promoters activity by 1.43-fold when compared to the scar in 101*_RFP_*. Error bars represent the 95% confidence intervals of the mean value computed on 3 clones.

The HSL-inducible system was characterized in terms of *S_cell _*as a function of exogenously added HSL concentrations. Results are reported in Figure 2 for all the copy number amounts. In all the considered situations, induction reaches a steady state value for HSL concentration >~10 nM. This result is consistent with previously measured induction curves of this HSL-inducible device [37,41]. Single-cell analysis was also performed to validate if all the cells of an induced culture respond to HSL and the results showed that the population was actually homogeneous for all the tested inducer concentrations (Supplementary Figure 2 in Additional file 1). Details about this analysis are reported in the Additional file 1 (Single-cell analysis). Table 4 reports the doubling time of the cultures in the different contexts. The coefficient of variation of the doubling time among different inductions of the HSL*_RFP _*device is less than 16%. The average doubling time of the cultures with the HSL*_RFP _*device varies only up to 1.4-fold (low copy condition) when compared to the culture bearing the reference device 101*_RFP _*or the IQ*_RFP _*device. On the other hand, doubling times vary more than 2-fold among different contexts without a specific trend with the copy number. The different antibiotics, plasmids and levels of the expressed heterologous genes may contribute to such unexpected difference in the doubling time values in the exponential phase, even if the chassis is the same. Note that, in our hands, the typical doubling time of the MG1655 strain in the same conditions is 38 (14%) min. As for the constitutive devices, also for the HSL-inducible system average values of *S_cell_*, measured at a full induction, were divided by the value measured in the single copy condition to obtain an indirect estimation of the plasmid copy number. Resulting ratios are 3.8, 28.5 and 42.1 for low, medium and high copy, respectively. They are quite different from the copy numbers found in the previous section for the two constitutive promoters, apparently yielding fewer DNA copies per cell in medium and high copy number conditions. Moreover, the high copy number value obtained for this system is much lower than previously reported for plasmids with the pUC19-derived pMB1 replication origin in cells grown at 37°C [39]. The growth conditions were the same among the tested cultures and the doubling times were comparable between HSL-inducible system and cultures bearing the constitutive promoters. Therefore, the difference in the estimated copy number is not reliable and more likely the observed effect is due to saturation in transcription and/or translation processes, caused by limited availability of polymerases or ribosomes in the medium and high copy number contexts. In order to enable the comparison of the HSL-inducible system activity among different experimental conditions, the RPU approach was used to express the strength of the luxI promoter relative to J23101 constitutive promoter as a reference [36]. RPUs have been reported to produce highly robust activity measurements even in different experimental conditions. Expressing the results in such standard units of measurement also enables the sharing of quantitative characterization results in the synthetic biology community. J23101 promoter showed an *S_cell _*variation in good agreement with the theoretical copy number of the plasmids used in this work (see Table 3) and the relative activity of the medium-strength promoter lacIQ could be effectively measured in RPUs in different copy number conditions, yielding highly reproducible results with an average value of 0.744 ± 0.05 (see Figure 3). Taken together, these results suggest that J23101 is a reliable reference for the computation of RPUs in all the copy number contexts. Figure 2 reports the resulting RPUs in the right vertical axis for each curve of the HSL-inducible device. RPU values at full induction (>10 nM) confirm that a saturation trend is present, as single and low copy conditions produce very similar values (RPUs ≊ 8.7), while the activity progressively decreases at medium (RPUs = 5) and high (RPUs = 2.8) copy contexts. In order to characterize the input-output function of the HSL-inducible device for each specific copy number condition, the curves were fitted with a Hill function, which well described the experimentally measured points. Estimated parameters of the functions are reported in Figure 2. Furthermore, to validate if the observed saturation trend was due to the specific reporter used, the full-induction activity of the HSL-inducible promoter was also measured via GFP (GFPmut3b) instead of RFP (mRFP1) in different copy number conditions. In addition, the GFP reporter device had a different RBS from the RFP device (BioBrick™BBa_B0032 instead of BBa_B0034). RPU results, shown in the Supplementary Figure 3 in Additional file 1 demonstrate an excellent accordance with the maximum activities reported in Figure 2, thus validating that the saturation trend in medium and high copy number contexts was actually due to the inducible device and not to the used reporter gene.

**Table 4:** Doubling times of the studied cultures.

Doubling time [min]				
	**HC**	**MC**	**LC**	**SC**

*HSL_RFP_*	127 (15%)	63 (7%)	131 (9%)	86 (5%)
101*_RFP_*	106 (11%)	59 (8%)	104 (9%)	86 (8%)
*IQ_RFP_*	102 (5%)	65 (6%)	92 (5%)	101 (17%)

Mean value and coefficient of variation (CV%, between brackets) were computed on 15 differently induced cultures in triplicate for *HSL_RFP _*and on 3 clones for 101*_RFP _*and *IQ_RFP_*.

### Measurement of the activity of the luxI promoter in different copy number conditions as a function of the activated complex

**Figure 4:**
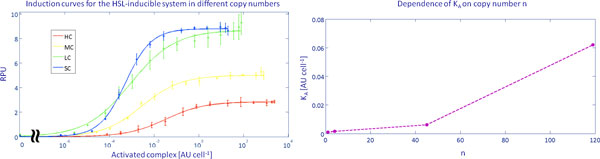
**Induction curves as a function of the activated complex and correlation between *K_A _*and *n***. Induction curves for the HSL-inducible system as a function of the activated complex HSL-LuxR in different copy number contexts (Panel A) and correlation between the estimated dissociation constant of HSL-LuxR complex and luxI promoter (*K_A _*parameter) and copy number (Panel B). The activity of the luxI promoter, in terms of RPUs, was studied in each copy number condition as a function of its actual inducer, i.e. the HSL-LuxR activated complex (*A*), indirectly measured by an ad-hoc mathematical model described in the Methods section.

In all the recombinant strains considered in this study, the DNA copy number of the luxR-expression cassette varies together with the luxI promoter-regulated RFP. The different availability of LuxR protein in the investigated conditions makes the HSL-RPU curves described in the previous section not easy to compare among the different copy number conditions, since the amount of the LuxR-HSL activated complex can be different even if the HSL concentration is the same. In order to study the strength of the luxI promoter in different copy number conditions, it is necessary to consider the induction curves as a function of the per-cell HSL-LuxR activated complex instead of the HSL concentration. In this way, correct comparisons can be performed to study the strength of the promoter at any induction entity. The mathematical model described in the Methods section was used to evaluate the quantity of the HSL-LuxR complex per-cell (indicated with *A*) and to express the system output, in terms of RPUs, as a function of the predicted intracellular level of *A*. The average DNA copy number indirectly measured in this study via RFP measurements in the constitutive devices (see Table 3) was used to predict the level of available LuxR proteins (X) at the steady state in each copy number condition. *K_A_*, *n_A _*and *α_lux _*parameters were estimated from HSL-RPUs experimental data. RPUs are reported in Figure 4A as a function of *A *for each copy number context. This figure enables the comparison of the promoter strength as a function of the actual activator level. Table 5 shows the estimated values of the model parameters. *V_max_*, reported in Table 5, depends on the RPUs reached for a full induction. *α_lux _*is proportional to *V_max _*and it decreases as the copy number increases. *n_A _*is similar among the conditions, even though for the single copy it is slightly higher than in the other cases. Moreover, it can be observed that the estimated *K_A _*values are correlated with the copy number. Figure 4B shows the *K_A _*parameter values as a function of the estimated copy number. Finally, the activated complex-RPUs induction curves show that the luxI promoter strength is comparable between single and low copy conditions for all the induction values, while medium and high copy curves show lower strength values than the single and low copy for all the induction levels.

**Table 5:** Estimated values for the *V*_*max*_, *α*_*lux*_, *K_A _*and *n_A _*parameters.

	*V_max _*[RPU]	*K_A _*[AU cell^-1^]	*n_A_*	*α_lux _*[AU min^-1 ^cell^-1^]
HC	2,84 (2%)	3,24e-2 (18%)	0,504 (8%)	2,11
MC	5,00 (1%)	2,87e-3 (13%)	0,502 (6%)	3,73
LC	8,68 (2%)	8,83e-4 (22%)	0,437 (8%)	5,70
SC	8,79 (1%)	5,13e-4 (6%)	0,747 (4%)	6,07

The coefficients of variation (CV%) are reported between brackets

## Conclusions

Because the design of genetic circuits which exhibit a predictable behaviour is the basis of the enormous potential of synthetic biology, the investigation of nonlinear phenomena and hard-to-predict engineered cell responses is essential to understand the systems under study and to avoid time- and cost-consuming strategies like trial-and-error approaches. Many variables, such as promoter strength, ribosome binding site efficiency and plasmid copy number, can play a crucial role in systems design. The optimization of a genetic circuitry can be achieved by tuning these elements, but the findings described in literature disclosed nonlinear effects [25,39]. The copy number of the DNA encoding the engineered genetic network is an important parameter for the regulation of gene dosage and many works reported its tuning to optimize biological circuits such as biosynthetic pathways [49]. However, as it happens with other biological parameters, the linearity of the output of a circuit may not be valid in many copy number ranges, yielding difficult-to-predict saturation effects. Hajimorad et al. studied the mRNA output of simple, independent gene expression cassettes as a function of the copy number, disclosing copy number ranges and conditions in which the superposition of the effects, typical of linear systems, is valid for the studied devices. Here, the aim of our work is to analyze the induction curves produced by an HSL-inducible system as a function of the copy number conditions, considering the fluorescence as the output. The studied system is composed by a luxR constitutive expression cassette and a RFP expression cassette regulated by the luxI promoter, which can be turned on by LuxR in presence of HSL with a concentration-dependent fashion. This device has been previously used in several works [14,37,41] and the improvement of its characterization in different copy number contexts can be useful for the rational design of expression systems and cell-cell communication networks involving HSL [16,17,19]. In this work, high, medium, low and single copy number conditions were considered. Medium-strength constitutive promoters contained in these plasmids or integrated in the genome exhibit activities that vary more than 100-fold from single to high copy number contexts, which demonstrates that copy number variation can be actually achieved with the used vectors. Moreover, for each constitutive promoter, the fold-change of the fluorescence of plasmid-bearing strains, relative to the fluorescence of the recombinant strain with the device in single copy (assuming a copy number = 1 by definition), is in accordance with the theoretical copy number of the used vectors. The strength of the HSL-inducible system was experimentally measured *in vivo *and expressed in RPUs, which are standard units for promoter activity evaluation proposed in literature to lower the variability among experiments. They also enable the sharing of the promoter characterization results in the synthetic biology community, as all the activity measurements are performed relative to the activity of a standard reference promoter (J23101), which gives RPUs = 1 by definition. Induction curves were obtained in different copy number conditions by exogenously adding HSL to the inducible system under investigation. A saturating trend in the maximum strength was depicted for higher copy number conditions, probably due to limited availability of polymerases or ribosomes which brings transcription/translation processes to saturation, as already reported in literature for other gene expression systems [39,42]. It is worth noting that, in addition to the luxI promoter strength, the presence of the luxR expression cassette may contribute to the saturating trend of the luxI promoter maximum relative activity in the higher copy number conditions by increasing the metabolic demand of the device. The same effect was seen for similar recombinant strains with the HSL-inducible device expressing a different fluorescent reporter gene (GFP instead of RFP), thus demonstrating that such phenomenon was not specific for the RFP gene. Characterization of the HSL-RPU transfer function was completed in each copy number context by fitting the curve with a Hill function. As they are expressed in standard units, the results produced here support the re-use of this inducible device, given the specific copy number in which it was characterized. However, these Hill curves do not directly represent the transfer functions of the luxI promoter, since it is not simply activated by HSL, but by the activator complex HSL-LuxR. The number of DNA copies of the luxR constitutive cassette varied in concert with the copy number of the rest of the device, so the copy number of this cassette leads to different availability of intracellular LuxR proteins in the different studied contexts. In order to obtain comparable transfer functions among different copy number conditions, an X-axis transformation on the HSL-RPU induction curves is required to express the promoter strength as a function of the concentration of the actual inducer. A model-based approach has been proposed to indirectly measure the HSL-LuxR activated complex concentration by predicting the intracellular availability of LuxR protein as a function of the expected copy number. This procedure allowed to express the model output (RPUs) as a function of the HSL-LuxR complex (called *A*). With the help of the mathematical model, the values of the promoter activity curves can be considered as a function of the correct inducer of the promoter, thus allowing comparisons of transcriptional strength in all the studied contexts. The relationship between *K_A _*(i.e. the dissociation constant between A and the luxI promoter) and the copy number shows correlation (see Figure 4), while the *K_m _*values found in the input-output induction curves simply as a function of HSL, showed no apparent correlation with the copy number (see Figure 2). However, it is important to note that the used model relies on parameters that have been indirectly estimated in this study (e.g. the plasmid copy number and intracellular concentration of LuxR protein). Moreover, this indirect estimation assumes that the luxR gene expression level per DNA copy (*α_tet_*) is constant for each copy number, neglecting any saturation effect in high copy number conditions. Even if more in-depth analysis of the biological systems under study can be performed to measure these parameters and to obtain more accurate relationships, the found behaviour for which the *K_A _*parameter value appears to correlate with the copy number is still valid, since the monotonically increasing trend of *K_A _*was confirmed even after a sensitivity analysis in which the (*α_tet_*) parameter was varied 2-fold (data not shown). Finally, a single-cell analysis was performed to improve the characterization of strains bearing the HSL-inducible device. From the literature, it is well known that in some inducible systems not all the cells among the population respond to the inducer, thus causing the simultaneous presence of induced/uninduced subpopulations [50,51]. On the contrary, results on the HSL-inducible device characterized in this work show that all the cells respond to the induction. This is a very interesting feature for the future usage of such HSL-inducible device in designing more complex circuits.

In conclusion, the characterization in standard, sharable units of a simple synthetic biological device has been performed and the input-output transfer function of the whole device has been reported for different copy number conditions. Even if the device is not significantly complex, nonlinear phenomena were evident in the higher copy number conditions. Moreover, the individual study of the activity of the inducible promoter present in the device was not trivial and it required a model-based approach to indirectly estimate hard-to-measure intracellular species, whose knowledge was necessary to completely characterize the system behaviour and to allow comparisons among different copy number contexts.

## List of abbreviations used

AU: arbitrary units; DNA: deoxyribonucleic acid; GFP: green fluorescent protein; HSL: N-3-oxohexanoyl-L-homoserine lactone; LB: Luria broth; mRNA: messenger ribonucleic acid; OD: optical density; PCR: polymerase chain reaction; RBS: ribosome binding site; RNA: ribonucleic acid; RFP: red fluorescent protein; RPU: relative promoter unit.

## Competing interests

The authors declare that they have no competing interests.

## Authors' contributions

SZ, LP and PM designed the experiments. SZ performed all the plasmid constructions and the validation experiments. GM performed the single-cell experiments. SZ, LP and PM analyzed the data and developed the mathematical model. LP drafted the manuscript and SZ, MGCDA and PM finalized it. All authors read and approved the final manuscript.

## Supplementary Material

Additional file 1 **Additional methods, results and supporting figures**. This file contains details about plasmid construction, validation of the measurement system and single cell-analysis. Three supplementary figures referenced in the main text are also included. Click here for file
