# Supplementary material for: Characterization of an inducible promoter in different DNA copy number conditions
Source: BMC Bioinformatics. 2012 Mar 28;13(Suppl 4):S11. doi: 10.1186/1471-2105-13-S4-S11 (PMC3314568; doi:10.1186/1471-2105-13-S4-S11)
Supplement: Additional file 1 — Additional methods, results and supporting figures. This file contains details about plasmid construction, validation of the measurement system and single cell-analysis. Three supplementary figures referenced in the main text are also included. [file 1471-2105-13-S4-S11-S1.doc]

**Plasmid construction**

Supplementary Table 1 lists all the plasmids used and/or assembled during this study. Plasmids containing the five final genetic systems used in quantitative experiments (HSL_RFP_, 101_RFP_ and IQ_RFP_, HSL_GFP_ and 101_GFP_) are in bold-type. pSCANSD is a pSCANS-derived vector (pSB2K3 in the Registry of Standard Biological Parts) which is propagated at high-copy number in TOP10 and recombinant strains bearing such plasmid vector were selected with Kanamycin at 50 mg/L during cloning [34].

|  | Plasmids |  |
| --- | --- | --- |
| Name | Description | BioBrick™ code |
| pMC-LAC_RFP_ | Hybrid promoter with lac operator sites with mRFP1 downstream in medium copy plasmid | pSB3K3(BBa_J04450)^a^ |
| pLC-ccdB | pUC19-derived pMB1 replication origin and ccdB toxin constitutive expression cassette in low copy vector | pSB4C5(BBa_I52002)^a^ |
| pФ80-ccdB | pUC19-derived pMB1 replication origin and ccdB toxin constitutive expression cassette in the conditional replication integrative vector | BBa_K300000(BBa_I52002)^b^ |
| pHC-RFP | Promoterless mRFP1 reporter device (i.e. with RBS upstream and transcriptional terminator downstream) in high copy plasmid | pSB1A2(BBa_I13507)^a^ |
| pHC-HSL | HSL-inducible device in high copy plasmid | pSB1A2(BBa_F2620)^c^ |
| **pHC-101_RFP_** | Standard reference constitutive promoter BBa_J23101 with mRFP1 expression device downstream in high copy plasmid | BBa_J61002(BBa_J23101)^a^ |
| pSCANSD-IQ | lacIQ constitutive promoter in pSCANS-derived plasmid | pSB2K3(BBa_I14032)^a^ |
| pSCANSD-IQ_RFP_ | lacIQ constitutive promoter with mRFP1 expression device downstream in pSCANS-derived plasmid | pSB2K3(BBa_J107012)^d^ |
| **pHC-IQ_RFP_** | lacIQ constitutive promoter with mRFP1 expression device downstream in high copy plasmid | pSB1A2(BBa_J107012)^d^ |
| **pHC-HSL_RFP_** | HSL-inducible mRFP1 expression system in high copy plasmid | BBa_J61002(BBa_F2620)^d^ |
| **pMC-HSL_RFP_** | HSL-inducible mRFP1 expression system in medium copy plasmid | BBa_J107055(BBa_F2620)^d^ |
| **pMC-101_RFP_** | Standard reference constitutive promoter BBa_J23101 with mRFP1 expression device downstream in medium copy plasmid | BBa_J107055(BBa_J23101)^d^ |
| **pMC-IQ_RFP_** | lacIQ constitutive promoter with mRFP1 expression device downstream in medium copy plasmid | pSB3K3(BBa_J107012)^d^ |
| **pLC-HSL_RFP_** | HSL-inducible mRFP1 expression system in low copy plasmid | BBa_J107056(BBa_F2620)^d^ |
| **pLC-101_RFP_** | Standard reference constitutive promoter BBa_J23101 with mRFP1 expression device downstream in low copy plasmid | BBa_J107056(BBa_J23101)^d^ |
| **pLC-IQ_RFP_** | lacIQ constitutive promoter with mRFP1 expression device downstream in low copy plasmid | pSB4C5(BBa_J107012)^d^ |
| pФ80-HSL_RFP_ | HSL-inducible mRFP1 expression system in the conditional replication integrative vector | BBa_J107057(BBa_F2620)^d^ |
| pФ80-101_RFP_ | Standard reference constitutive promoter BBa_J23101 with mRFP1 expression device downstream in the conditional replication integrative vector | BBa_J107057(BBa_J23101)^d^ |
| pФ80-IQ_RFP_ | lacIQ constitutive promoter with mRFP1 expression device downstream in the conditional replication integrative vector | BBa_K300000(BBa_J107012)^d^ |
| **pHC-101_GFP_** | Standard reference constitutive promoter BBa_J23101 with GFPmut3b expression device downstream in high copy plasmid | pSB1A2(BBa_K173001)^b^ |
| **pHC-HSL_GFP_** | HSL-inducible GFPmut3b expression system in high copy plasmid | pSB1A3(BBa_T9002)^a^ |
| **pMC-HSL_GFP_** | HSL-inducible GFPmut3b expression system in medium copy plasmid | pSB3K3(BBa_T9002)^d^ |
| **pMC-101_GFP_** | Standard reference constitutive promoter BBa_J23101 with GFPmut3b expression device downstream in medium copy plasmid | pSB3K3(BBa_K173001)^d^ |
| **pLC-HSL_GFP_** | HSL-inducible GFPmut3b expression system in low copy plasmid | pSB4C5(BBa_T9002)^d^ |
| **pLC-101_GFP_** | Standard reference constitutive promoter BBa_J23101 with GFPmut3b expression device downstream in low copy plasmid | pSB4C5(BBa_K173001)^d^ |
| ^a^ taken from the Registry DNA Distribution 2009  ^b^ previously constructed in our laboratory  ^c^ given by the iGEM Headquarters, Massachusetts Institute of Technology, Cambridge, USA  ^d^ constructed in this study  The BioBrick™ code of the plasmids includes vector name with insert name in brackets.  BBa_J61002, BBa_J107055, BBa_J107056 and BBa_J107057 are identical to pSB1A2, pSB3K3, pSB4C5 and BBa_K300000, respectively, but they have a promoterless mRFP1 expression device between SpeI and PstI in the BioBrick™ cloning site suffix. pSB1A3 is identical to pSB1A2, but it has an additional transcriptional terminator downstream of the cloning site.  The prefix pHC- indicates a high copy vector backbone (pSB1A2, pSB1A3 or BBa_J61002), pMC- indicates a medium copy vector backbone (pSB3K3 or BBa_J107055), pLC- indicates a low copy vector backbone (pSB4C5 or BBa_J107056), pФ80- indicates an integrative conditional replication vector (BBa_K300000 or BBa_J107057) and pSCANSD- indicates a variable copy number vector backbone (pSB2K3). | | |

pHC-HSL_RFP_ was obtained by assembling the promoterless RFP-containing insert of pHC-101_RFP_ digested with SpeI-PstI to pHC-HSL digested with SpeI-PstI. The insert of pHC-RFP was digested with XbaI-PstI and assembled to pSCANSD-IQ, digested with SpeI-PstI, to obtain pSCANSD-IQ_RFP_. pHC-IQ_RFP_ was obtained by assembling the insert of pSCANSD-IQ_RFP_ digested with EcoRI-PstI to pHC-101_RFP_ vector backbone, digested with EcoRI-PstI, thus eliminating its original insert. All the other plasmids were obtained by assembling the inserts of pHC-HSL_RFP_, pHC-101_RFP_, pHC-IQ_RFP_, pHC-HSL_GFP_ or pHC-101_GFP_, digested with EcoRI-PstI, to the appropriate vector backbones (from pMC-LAC_RFP_, pLC-ccdB or pФ80-ccdB plasmids) digested with EcoRI-PstI to eliminate their original inserts.

**Validation of the measurement system**

The main purpose of this work is to study nonlinear effects in a biological system, measuring suitable outputs. It is then crucial to preliminarily verify that the measurement system itself, i.e. the TECAN Infinite F200, does not introduce nonlinearities in the measurements, at least in the range of the working conditions of interest. To this aim, the following ad-hoc experiment was performed: a fluorescent culture of MG1655 bearing pHC-HSL_RFP_, grown to OD600=0.5 in selective M9 supplemented medium and induced with 100 nM of HSL, was serially diluted in fresh medium and absorbance (OD_600_) and fluorescence (RFP) were statically measured (see the main text, Methods section, for technical details). After a background subtraction (see the main text for details), OD_600_ measurements were plotted against the dilution factor and RFP was plotted against the OD_600_. The resulting curves are shown in the Supplementary Fig. 1.


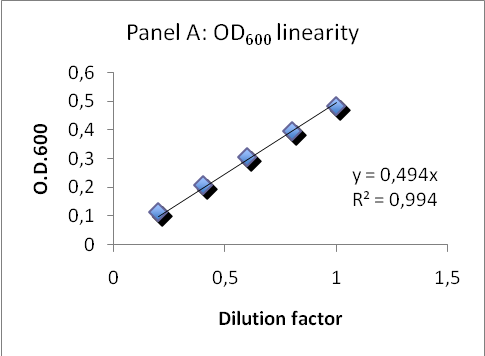

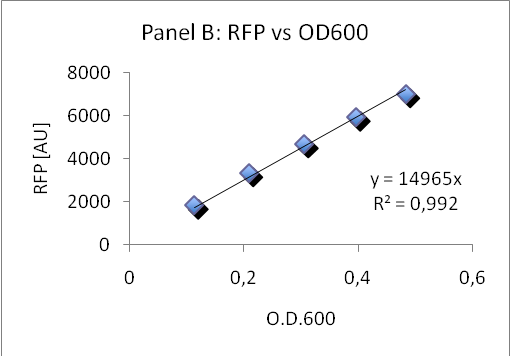


**Supplementary Figure 1**. *Linearity between OD600 and dilution factor (panel A) and between RFP and OD_600_ measurements (panel B) for a culture of MG1655 bearing pHC-HSL_RFP_ induced at 100 nM. Diamonds represent experimental data points and continuous line represents the linear regression line. AU indicates arbitrary units of RFP.*

Both absorbance and fluorescence measurements show a linear dependence from the dilution (R^2^>0.99 for both of them). Note that in the quantitative experiments performed in this work measurements were taken only in the exponential growth phase, which ends up at OD600<0.2 for all the tested cultures. Therefore, in this range, the measurement system does not introduce nonlinear effects during data acquisition. Similar experiments were done with GFP-expressing cells and they demonstrated the absence of nonlinearities also for GFP fluorescence detection (data not shown).

Moreover, in order to verify that changes in the fluorescence gain do not introduce nonlinear effects, the above procedure was repeated at different gain factors. Results confirm the linear relationships for all the investigated fluorescence acquisition gains (data not shown).

**Single-cell analysis**

Homogeneity of reporter expression level throughout the population was studied in order to assess if all the bacterial cells can respond to induction by HSL. Recombinant strains bearing the RFP- or GFP-expressing HSL-inducible systems in medium copy number were induced via three different HSL concentrations and analyzed through fluorescence microscopy or flow cytometry. Supplementary Fig.2 reports representative microscopy images and fluorescence histograms. Supplementary Fig.2A shows that all the cells detected in the bright-field express red fluorescence upon induction for the HSL-inducible device with 1 nM, 10 nM and 10 µM of HSL. Uninduced bacteria show a much lower per-cell fluorescence. Such low fluorescence intensity is not seen in any cell when cultures are induced. Fluorescence histograms in the Supplementary Fig.2B show that GFP distributions are all unimodal, with their average value increasing with the HSL concentration, as expected from the induction curves (see Fig.2 in the main text). As observed in the microscopy images, in the histograms of the three induced cultures no significant amount of the cell population expresses the typical fluorescence intensity seen in the GFP histogram of the uninduced culture.

The overall results of microscopy and flow cytometry are in accordance and demonstrate that induced bacterial populations homogeneously respond to HSL.


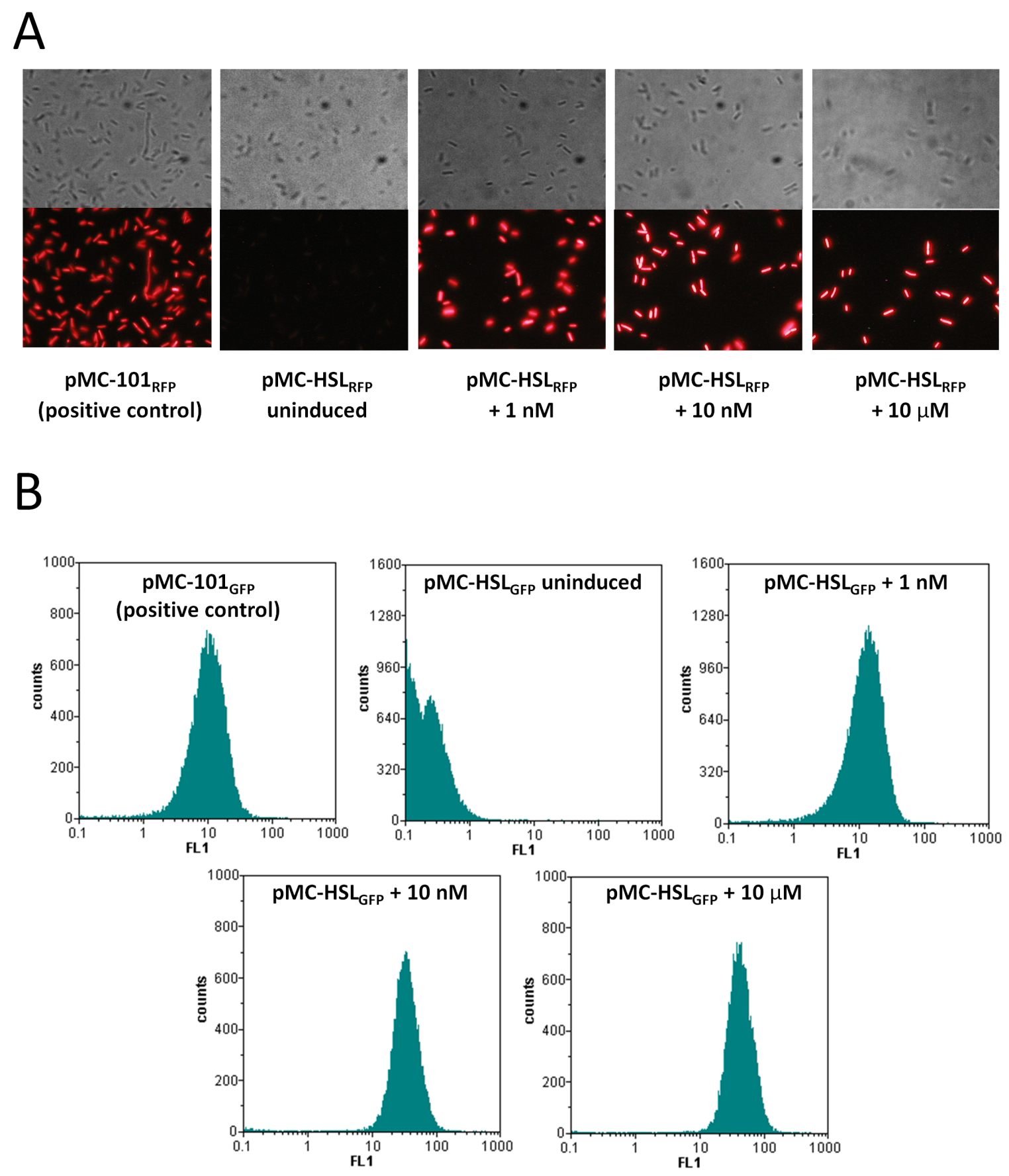


**Supplementary Figure 2**. *A) Bright-field and fluorescence images acquired in the microscopy analysis for the RFP-expressing HSL-inducible device in medium copy. All the fluorescence images have been acquired with the same exposition time of 1.3 s. B) Fluorescence histograms acquired in the flow cytometric analysis for the GFP-expressing HSL-inducible device in medium copy. In all the plots, the x-axis represents the fluorescence intensity (in arbitrary units of GFP) and the y-axis represents the cell count.*

**
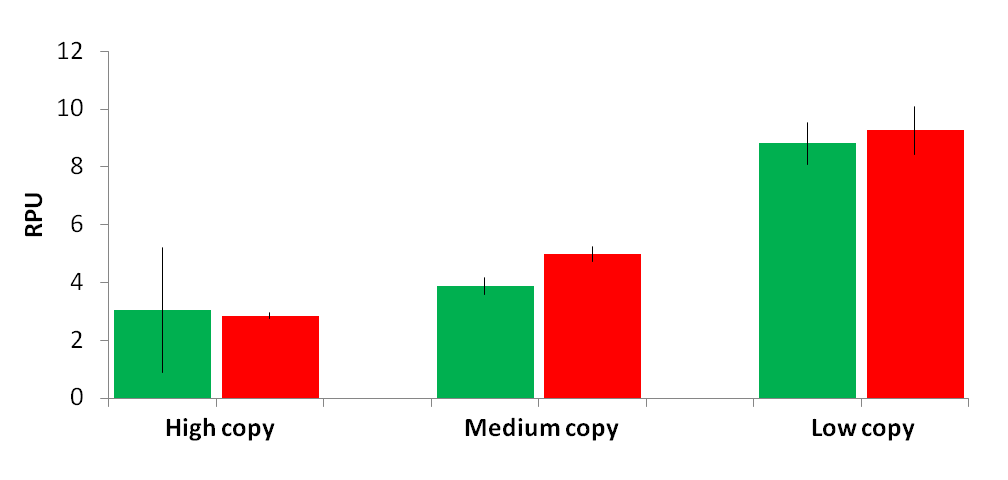
**

**Supplementary Figure 3**. *Comparison of the relative activity of the HSL-inducible device at full induction (i.e. induced with 10 µM of HSL) measured via GFP (green bars) or RFP (red bars) reporter devices. Error bars represent the standard deviation of three independent experiments.*
